# POLE2 facilitates the malignant phenotypes of glioblastoma through promoting AURKA-mediated stabilization of FOXM1

**DOI:** 10.1038/s41419-021-04498-7

**Published:** 2022-01-17

**Authors:** Peng Zhang, Xu Chen, LingYun Zhang, Dan Cao, Yong Chen, ZhengQian Guo, Jian Chen

**Affiliations:** 1 Department of Neurosurgery of the First Affiliated Hospital of Zhengzhou University, Zhengzhou, No.1 Jianshe East Road, Zhengzhou City, Henan Province China; 2 Department of Neurosurgery, Tongji Hospital, Tongji Medical College, Huazhong University of Science and Technology, No. 1095 Jiefang Ave, Wuhan City, Hubei Province China; 3 Department of Thyroid and Parathyroid Surgery, West China Hospital, Sichuan University, No. 37 Guoxue Alley, Chengdu City, Sichuan Province China

**Keywords:** CNS cancer, Cell biology, Molecular biology

## Abstract

Glioblastoma (GBM) is a type of brain cancer with high morbidity and mortality worldwide. The clinical significance, biological roles, and underlying molecular mechanisms of DNA poly ε-B subunit (POLE2) in GBM were investigated in the study. Firstly, the Cancer Genome Atlas (TCGA) database found that POLE2 was highly expressed in GBM. Immunohistochemistry (IHC) results further confirmed that POLE2 was abnormally elevated in GBM. In addition, loss-of-function assays revealed that POLE2 knockdown could inhibit the malignant behaviors of GBM, especially reduce cell viability, weaken cell clone formation, enhance the sensitivity of apoptosis, restrain migration and inhibit epithelial-mesenchymal transition (EMT) in vitro. In vivo experiments further clarified the suppressive effects of reduced POLE2 expression on tumors. Mechanically, POLE2 knockdown promoted the ubiquitination as well as reduced the stability of Forkhead transcription factor (FOXM1), which is a known tumor promotor in GBM, through Aurora kinase A (AURKA). Moreover, the knockdown of FOXM1 could weaken the promoting effects of POLE2 on malignant behaviors of GBM. In conclusion, our study revealed crucial roles and a novel mechanism of POLE2 involved in GBM through AURKA-mediated stability of FOXM1 and may provide the theoretical basis of molecular therapy for GBM.

## Introduction

Glioma is one of the most common primary central nervous system tumors in adults, accounting for more than 70% of malignant brain tumors [1]. In general, gliomas can be divided into oligodendrogliomas, meningiomas, and astrocytomas [2]. According to histopathological characteristics, the World Health Organization (WHO) further divides astrocytomas into grade I (astrocytoma) and grade II (diffuse astrocytoma), grade III (anaplastic astrocytoma), and grade IV (pleomorphic glioblastoma) (GBM) [3]. At present, the traditional treatment of glioma includes surgical resection, radiotherapy, and temozolomide (TMZ) adjuvant chemotherapy [4]. Among them, grade I and II have slow growth, poor invasiveness, good prognosis, and sensitivity to treatment [5]. Unfortunately, GBM are highly invasive and lethal, which are easy to relapse and have poor therapeutic effects due to resistance to chemotherapy and radiotherapy [6]. In the world, the incidence and mortality of GBM are in the forefront, showing an upward trend year by year [7]. In addition, GBM has a poor prognosis, with an overall survival of less than 15 months after diagnosis [8]. The development of more effective and accurate therapies relies on the exploration of the molecular mechanisms of GBM. Therefore, it is of great significance to identify potential molecular targets related to the behaviors and mechanism of GBM.

The human genome contains at least 15 DNA polymerase for genome replication, DNA repair, and cell cycle control [9]. Eukaryotic DNA polymerase epsilon (DNA poly ε) was first isolated from Saccharomyces cerevisiae in 1970 [10]. DNA poly ε consists of four subunits POLE, POLE2, POLE3, and POLE4, of which POLE (A subunit) is the largest subunit and POLE2 (B subunit) is the second largest with a molecular weight of 59 kDa [11]. These subunits are involved in synthesis regulation and co-factor binding [12]. Somatic exonuclease domain-mutations in POLE have been identified in colorectal cancer and endometrial cancer patients, and showed association with hypermutability and microsatellite-stability [13, 14]. In recent years, numerous evidences indicated that POLE2 is abnormally overexpressed in lymphoma [15], cervical cancer [16], bladder cancer [17], lung adenocarcinoma [18], breast cancer [19], and colorectal cancer [20]. Moreover, Wu et al., suggested that the high expression of POLE2 is a biomarker associated with poor survival and prognosis of squamous cell lung cancer, and negatively correlated with immune infiltration [21]. The latest report demonstrated that knockdown of POLE2 can inhibit the tumor progression of esophageal squamous cells [22]. So far, the function and regulation of POLE2 in GBM have not been explored. In this context, the clinical significance, biological roles, and downstream regulatory mechanism of POLE2 in GBM were investigated.

## Materials and methods

### Immunohistochemical (IHC) analysis

The tissue microarray of human survival glioma (Outdo Biotech Company, Shanghai, China) has a total of 180 points, including detailed pathological data such as gender, age, pathological grade, and so on. All patients signed informed consent to use clinical data for research purposes. Briefly, deparaffinize and rehydrate by immersing the tissue microarray through xylene and ethanol. After the tissue sections were washed twice with 1% animal serum (Thermo Fisher Scientific, California, USA, Cat. No. 1921005PJ) in PBS with 0.4% Triton X-100 (PBS-T), the primary antibody POLE2 (Table S1) diluted in 1% animal serum in PBS-T and incubate at room temperature for 2 h. Continue the incubation with secondary antibody overnight at 4 °C in a humidified chamber. The tissue sections were stained with DAB solution and hematoxylin in turn. All tissue chips were photographed with microscopic, viewed with ImageScope and CaseViewer. Notably, high or low expression of POLE2 was determined by the median of IHC scores of all tissues.

The mice tumor tissues were fixed with 10% formalin, immersed in xylene and ethanol in turn. After tumor tissues were blocked with 3% PBS-H_2_O_2_, incubated with anti-Ki67 and HRP goat anti-rabbit IgG (The detail was listed in Table S1), respectively. Finally, slides were stained by Hematoxylin (Cat. No. 517-28-2, Sigma-Aldrich^®^, St. Louis, Missouri, USA) and Eosin (Cat. No. 548-24-3, Sigma-Aldrich^®^, St. Louis, Missouri, USA) as well as examined at ×200 objective lens microscopic.

### Cell culture

The U87 [23] and U251 [24] cell lines (Cell Bank of Chinese Academy of Sciences, Shanghai, China) are commonly used as experimental models of GBM, which were tested for mycoplasma contamination. HEK 293T cells, is an adhesion-dependent epithelialize-like cell, often referred to as “tool cells”, used for lentivirus packaging production and titer determination, cell transfection. All the cells were cultivated in DMEM medium (Gibco, life technologies, California, USA) supplemented with 10% fetal bovine serum and 100 mg/mL streptomycin plus 100 UI/mL of penicillin (Gibco, life technologies, California, USA) in the atmosphere of 5% CO_2_ at 37 °C.

### Lentiviral shRNA vector construction and cell infection

Interfering sequences containing the target gene were synthesized using the POLE2, FOXM1, and AURKA sequence as template, which were directly connected to the lentiviral vector BR-V-108 (Bio Sci Res, Shanghai, China). Meanwhile, the amplified sequence of POLE2 was linked to the lentiviral vector. The lentiviral plasmid containing the target sequence was transfected using Lipofectamine^®^ 2000 (Invitrogen; Thermo Fisher Scientific, California, USA) at a MOI (multiplicity of infection) of 10 to infect U87 and U251 cells, respectively. After 72 h, the expression of green fluorescent protein (GFP) was observed under the fluorescence microscope (Cat. No. IX71, OLYMPUS, JPN).

### RNA isolation and qPCR

After the U87 and U251 cells were collected, they were cleaved by Trizol for total RNA extraction. The concentration and quality of the extracted RNA were analyzed and determined by Nanodrop 2000/2000 C spectrophotometer. The cDNA was obtained by reverse transcription using Promega M-MLV kit. The qPCR was accomplished with the SYBR Green PCR kit (Thermo Fisher Scientific, California, USA) and quantification was analyzed by the method of 2^-ΔΔCq^. Notably, the primers were listed in Table S2, where GAPDH as an internal control.

### Western blotting (WB) analysis

After total proteins of U87 and U251 cells were extracted, quantified by BCA protein assay kit (Cat. No. A53227, Thermo Fisher Scientific, California, USA). CHX (0.2 mg/mL) refers to Cycloheximide blocking protein biosynthesis to study the half-life of FOXM1 and AURKA proteins over time (0–8 h). Same for the use of the proteasome inhibitor MG-132 (20 μM) to inquire about the protein degradation. Equivalent amount of protein was separated through 10% SDS-polyacrylamide gel electrophoresis (SDS-PAGE) and transferred to polyvinylidene fluoride (PVDF) film at 4 °C. The protein was incubated with primary antibody and secondary antibody (antibody information was listed in Table S1) in turn at 4 °C for 3 h. The immune response was visualized with the Amersham ECL + plusTM Western Blot system, and the blots were imaged by luminescent image analyzer.

### Co-Immunoprecipitation (Co-IP) assay

After the U251 cells were lysed, the protein was obtained and the concentration was measured by BCA protein detection kit (Thermo Fisher Scientific, California, USA). The protein–protein interactions were analyzed as described in the literature [25].

### MTT cell viability assay

U87 and U251 cells were cultured in 96-well plates at 2000 cell/well density for 24 h. The 20 μL of 5 mg/mL MTT (Genview, Cat. No. JT343, Shanghai, China) was added 4 h before the termination of the culture. After 4 h, the culture medium was completely absorbed and 100 μL of DMSO was added to dissolve formazan granules. After 2–5 min of oscillation, OD value at 490/570 nm was detected by microplate reader (Cat. No. M2009PR, Tecan infinite M200, Switzerland) for 5 days.

### Colony formation assay

U87 and U251 cells were cultured in 96-well plates at 2000 cell/well density for 14 days. Subsequently, the cells were fixed with 1 mL 4% paraformaldehyde in each well for 60 min and washed with PBS. Next, cells were stained with GIEMSA 500 μL for 20 min, washed several times with ddH_2_O and dried. Cell clones were photographed and counted under a fluorescence microscope.

### Transwell assay

After 18 h of U87 and U251 cells culture in 24-well plates at a density of 1 × 10^5^ cell/well, 100 μL of cell suspension was placed in each chamber (3422 corning). The 600 μL medium containing 30% FBS was added to the lower chamber, and 100 μL serum-free medium was added to the up chamber. After incubation for 2 h, the culture medium in the chamber was removed and transferred to the lower chamber. After 24 h of culture, the medium was removed by inverting the chamber on the absorbent paper, and the non-metastatic cells were gently removed with a cotton swab. The 400 μL staining solution was added to the 24-well plate for staining the transferred cells for 20 min. Subsequently, the chamber was washed with water for several times to dry. The 10% acetic acid was added to detect OD570 and the film was photographed under a microscope.

### Wound-healing assay

U87 and U251 cells were cultured into 6-well plates (100 μL/well) at a density of 4000 cells per well. The cells were eluted with PBS, fixed with 3.7% paraformaldehyde (Corning) for 15 min, stained with 1% crystal violet for 10 min. Wound healing was observed at 0, 8, and 72 h under a microscope for image acquisition and Image J software (National Institutes of Health) was used to quantify the distance (μm) between the scratches.

### Cell apoptosis analysis by Flow cytometry

U87 and U251 cells were cultured in 6-well plates at 2 mL/well for 5 days and centrifuged. Then, the cell precipitation was washed successively with the 4°C pre-cooled D-Hanks (pH = 7.2–7.4) and 1×binding buffer. Annexin V-APC (cat. no. 88-8007-74, eBioscience, Thermo Fisher Scientific, California, USA) 10 μL was added to stain cells at 37 °C in the dark for 10–15 min. The number of apoptotic cells was detected using the FACSCanto II flow cytometer (cat. no. Guava easyCyte HT, Millipore, Massachusetts, USA) after adding 400-800 μL of 1×binding buffer.

### Human apoptosis antibody array

After total proteins of U251 cells were extracted, quantified by BCA protein assay kit (Cat. No. A53227, Thermo Fisher Scientific, California, USA). The protocol was operated according to the instruction of the human apoptosis antibody array membrane (Cat. No. ab134001, Abcam, Cambridge, UK). Finally, the array membranes were exposed in the chemiluminescence imaging system.

### Xenograft mouse tumor model

The animal experiments were in accordance with the Guide for Care and Use of Laboratory animals (NIH publication number 85-23, revised at 1996) and approved by the Ethics Committee of the First Affiliated Hospital of Zhengzhou University. Twenty 4-week-old female BALB/ C nude mice (Lingchang Biotechnology, Shanghai, China) were randomly divided into two groups of shCtrl (negative control, *n* = 10) and shPOLE2 (POLE2 knockdown, *n* = 10). The U87 cells were prepared into cell suspension and subcutaneously injected with 200 μL at 4 × 10^6^ cells/mL to the right forearm of each mouse. About a week later, the mice were anesthetized by intraperitoneal injection of 0.7% pentobarbital sodium at 10 μL/g and placed in the living imaging system (Cat. No. LB983, Berthold Technologies, Germany) for imaging and fluorescence observation. Tumor size and mice weight were measured every other day until 26 days after subcutaneous injection. The mice were physically killed and the tumors were removed, weighed and photographed.

### Human Gene Chip

The U251 cells RNA with complete fragments and high purity was used for the analysis of molecular mechanism through Affymetrix human Gene Chip Prime View combined with Affymetrix Scanner 3000 scan (Affymetrix, Santa Clara, CA, USA). The Human Gene Chip technology has been used to detect differentially expressed genes (DEGs) in different groups of shPOLE2 and shCtrl in U251 cells. The volcano plot and hierarchical clustering of the shPOLE2 and shCtrl in U251 cells were presented by the DEGs with criterion of |Fold Change|≥1.3 and false discovery rate (FDR) ≤ 0.05. Furthermore, the significant enrichment of DEGs in canonical pathways as well as disease and function were explored based on Ingenuity Pathway Analysis (IPA) (Qiagen, Hilden, Germany).

### Statistical analysis

Statistical analyses were conducted by SPSS 19.0 with GraphPad Prism 8.0 software and data were expressed as the mean ± standard deviation. The statistical significance between different groups was accomplished by independent Student’s *t* test and *P* < 0.05 was considered statistically significant. The correlation between POLE2 expression and clinic characteristics of GBM was evaluated by Mann–Whitney *U* analysis. Survival curves were obtained by the Kaplan–Meier method, and differences in survival rates were assessed by the log-rank test. All statistical tests were two-tailed and values of *P* < 0.05 were considered statistically significant.

## Results

### POLE2 is highly expressed in human GBM

**Fig. 1:**
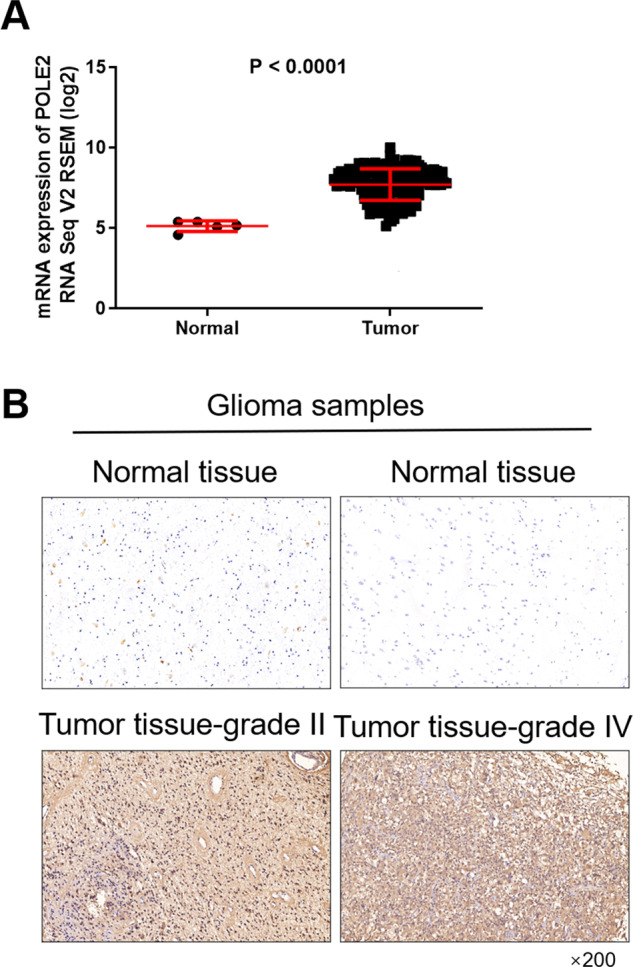
POLE2 is highly expressed in human GBM. **A** The mRNA expression of POLE2 in GBM samples (169 cases) and normal samples (5 cases) was compared from TCGA-GBM database. **B** The representative picture of the expression level of POLE2 in normal tissues and tumor tissues with different grades was detected by immunohistochemistry (IHC). The magnification is 200.

Firstly, we found that the mRNA expression of POLE2 in tumor samples (169 cases) was significantly higher compared with the normal samples (5 cases) from the Cancer Genome Atlas (TCGA) database (Fig. 1A). Based on the database, we further analyzed the correlation analysis between POLE2 expression and the survival probability of patients with GBM. Perhaps due to the sample size of the database, there is no significant correlation between them (Fig. S1A). In order to further clarify the expression level of POLE2 in GBM, we performed IHC staining analysis in normal brain tissue [15] and tumor tissue (165) in clinical GBM patients. According to the scores of IHC staining, we defined POLE2 as a high expression if it is greater than the median, otherwise as low expression. Consistently, the results of IHC staining showed that the signal intensity of POLE2 in GBM tissues was stronger than that in adjacent normal tissues (Fig. 1B). The representative image of IHC staining indicated that the higher the pathological grade, the higher the expression of POLE2 in the tumor tissue. The relationships between POLE2 expression and clinic characteristics of GBM were summarized in Table 1. A total of 75 (45.4%) cases showed high levels of POLE2 expression (score 5–12). We further suggested that the POLE2 expression was positively correlated with the stage and recurrence of GBM (*P* < 0.001, Table 1). However, no statistically significant correlation was observed between POLE2 expression and other clinicopathologic features, such as age and gender.

**Table 1:** Correlation analysis between POLE2 expression level and clinic characteristics of GBM.

Features	No. of patients	POLE2 expression		*P* value
		Low	High	
All patients	165	90	75	
Age				0.057
<42	86	53	33	
≥42	79	37	42	
Gender				0.126
Male	104	52	52	
Female	61	38	23	
Stage				<0.001
I–II	93	83	10	
III–IV	72	7	65	
Recurrence				<0.001
Yes	91	28	63	
No	74	62	12	

### POLE2 knockdown inhibits the proliferation and migration of GBM cells in vitro

**Fig. 2:**
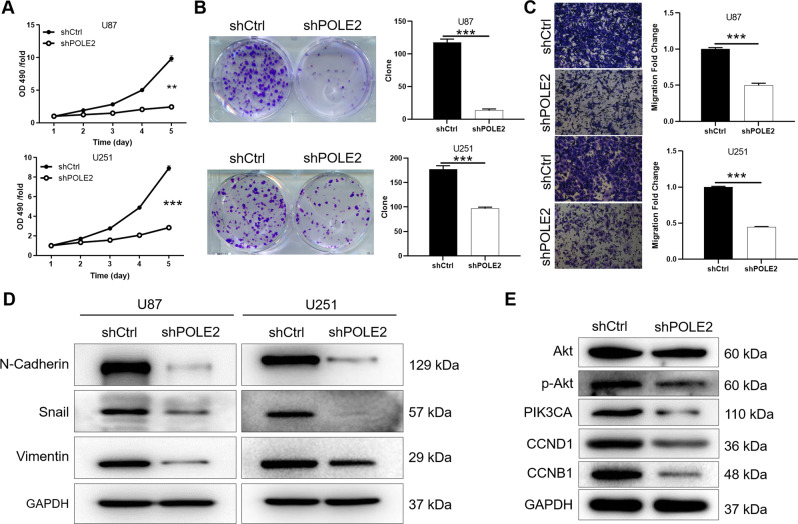
POLE2 knockdown inhibits the viability and migration of GBM cells in vitro. **A** MTT cell viability assay was employed to show the effects of POLE2 on U87 and U251 cells. **B** Colony-forming ability of U87 and U251 cells was detected in shPOLE2 and shCtrl groups. **C** The U87 and U251 cell migration ability was accessed by Transwell assay. **D** The protein expression of N-cadherin, Vimentin, and snail of U87 and U251 cells was measured by WB. **E** The downstream protein expression of Akt, p-Akt, PIK3CA, CCND1, and CCNB1 of U251 cells was measured by WB. The representative images were selected from at least three independent experiments. Data were shown as mean ± SD. **P < 0.01, ***P < 0.001.

In order to further clarify the biological function of POLE2 in GBM, knockdown of POLE2 in U87 and U251 cells were constructed. More than 80% of the cells showed GFP indicating that the lentiviruses shCtrl (negative control) and shPOLE2 successfully infected (Fig. S1B). Subsequently, the mRNA expression of POLE2 in U87 and U251 cells in shPOLE2 group was significantly lower than that in shCtrl group (Fig. S1C). Moreover, the WB results of U87 and U251 cells indicated that POLE2 bands in shPOLE2 was weaker than that in the control group (Fig. S1D). Therefore, POLE2 was knocked down in U87 and U251 cells for the detection of cell function. The results of MTT assay showed that the OD490 value of shPOLE2 group in U87 and U251 cells were lower than that of the control group, indicating that the downregulation of POLE2 resulted in a significant decrease in cell viability (Fig. 2A). Consistently, knockdown of POLE2 contributed to an obvious decrease in the number of colonies in GBM cells (*P* < 0.001) (Fig. 2B). Transwell results further indicated that the cell migration ability of shPOLE2 group was inhibited compared with shCtrl group (*P* < 0.001) (Fig. 2C). In view the fact that epithelial-mesenchymal transition (EMT) is a developmental procession that induces invasion and metastasis in various types of tumors [26]. During EMT, N-cadherin, Snail, and Vimentin are the most frequent detected epithelial and mesenchymal markers, respectively [27]. Thus, our results indicated that knockdown of POLE2 led to the downregulation of N-cadherin, Snail, and Vimentin of U87 and U251 cells (Fig. 2D). Furthermore, EMT process involves multiple regulatory mechanisms, including phosphorylated Akt serine/threonine kinase (p-Akt) activation and cyclin alteration [28, 29]. The present study showed that knockdown of POLE2 resulted in the downregulation of p-Akt, PIK3CA, G1 cyclin D1 (CCND1), and cyclin B1 (CCNB1) (Fig. 2E). Collectively, knockdown of POLE2 through EMT inhibited the malignant behaviors of GBM cells.

### POLE2 knockdown enhances the apoptosis of GBM cells in vitro

**Fig. 3:**
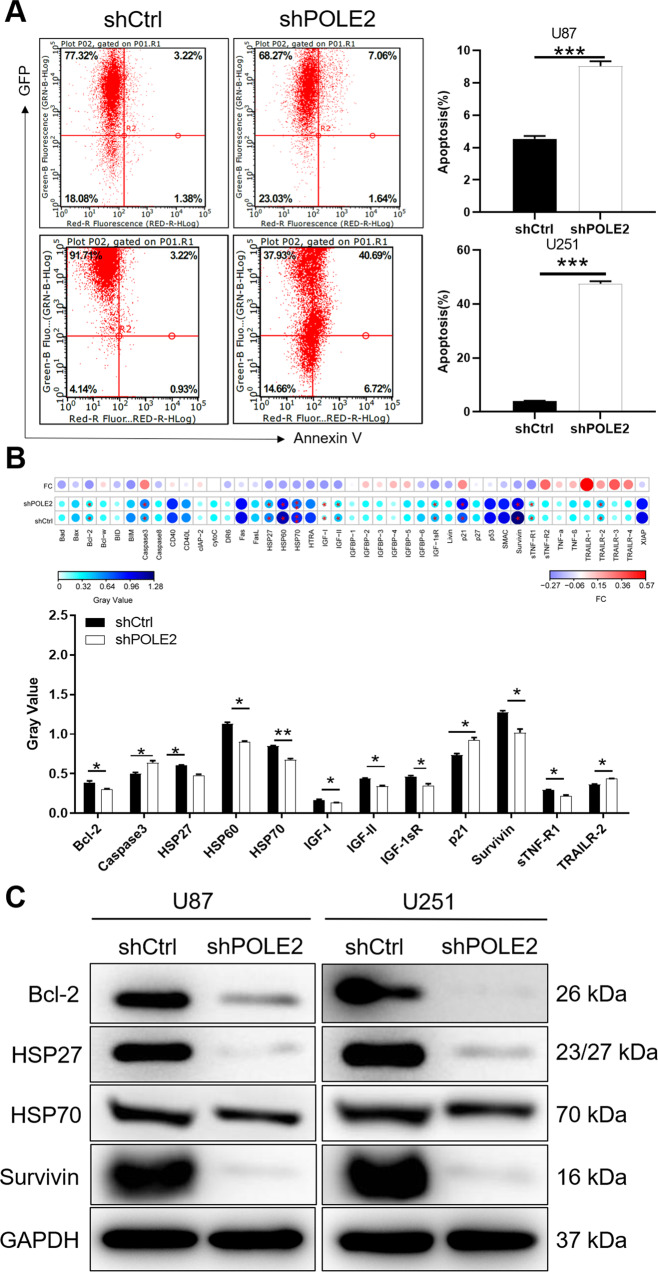
POLE2 knockdown enhances apoptosis of GBM cells in vitro. **A** Flow cytometry was performed to detect cell apoptosis of U87 and U251 cells with or without POLE2 knockdown. The representative images were selected from at least three independent experiments. **B** Human apoptosis antibody array was utilized to analyze the regulatory ability of POLE2 on expression of apoptosis-related proteins in U251 cells. **C** The protein levels of Bcl-2, HSP27/70, and Survivin in U87 and U251 cells with or without POLE2 knockdown were further analyzed by WB. Data were shown as mean ± SD. *P < 0.05, ***P < 0.001.

In addition, the cell apoptosis of U87 and U251 cells following POLE2 knockdown was measured by flow cytometry. The results presented that the percentage of apoptosis was increased in shPOLE2 group compared with shCtrl group (*P* < 0.001), suggesting that knockdown of POLE2 enhanced apoptosis of U87 and U251 cells (Fig. 3A). Collectively, the results provided evidence that downregulation of POLE2 significantly induced the apoptosis of GBM cells, and played important roles in the regulation of cell viability. To further explore the molecular mechanism underlying cell apoptosis accelerated by POLE2 knockdown, we used the human apoptosis antibody array membrane to simultaneously detect the differential expression of 43 apoptosis-related proteins in shCtrl and shPOLE2 groups. As illustrated in Fig. 3B, knockdown of POLE2 in U251 cells resulted in the abnormal expression of apoptosis-related proteins in human apoptosis signal pathway. Specifically, the expression of Caspase3, p21, Tumor necrosis factor (TNF)-related apoptosis-inducing ligand (TRAIL) receptors (TRAILR)-2 was upregulated, whereas the expression of Bcl-2, heat shock protein 27 (HSP27), heat shock protein 60 (HSP60), heat shock protein 70 (HSP70), insulin-like growth factor-I (IGF-I), insulin-like growth factor-II (IGF-II), IGF system components (IGF-1sR), soluble tumor necrosis factor receptor R1 (sTNF-R1) and Survivin was downregulated. Consistently, results of WB analysis further showed that the protein levels of Bcl-2, HSP27/70, and Survivin were downregulated in GBM cells with POLE2 knockdown (Fig. 3C).

### POLE2 knockdown suppresses GBM growth in vivo

**Fig. 4:**
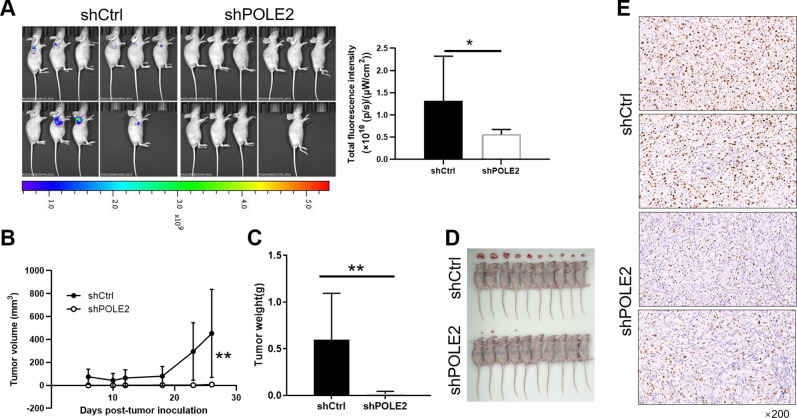
POLE2 knockdown suppresses GBM growth in vivo. **A** In vivo imaging was performed to evaluate the tumor burden in mice of shPOLE2 and shCtrl groups post tumor-inoculation **B**–**C** U87 cells with or without POLE2 knockdown, the volume (**B**) and weight (**C**) of tumors formed in mice was measured and calculated at indicated time intervals. **D** photo of the removed tumors was taken post tumor-inoculation. **E** The Ki67 level in tumors removed from mice was detected by IHC as a representation of tumor growth. Data were shown as mean ± SD. *P < 0.05, **P < 0.01.

The xenograft mice tumor models were established to further verify the role of POLE2 in regulating GBM cells in vivo. As illustrated in Fig. 4A, the fluorescence intensity of tumors in shPOLE2 group was obviously weaker than that of shCtrl, which preliminarily indicated that the downregulation of POLE2 could reduce the ability of tumor formation. Furthermore, the tumor volume of mice in the shPOLE2 group was remarkably smaller than that of mice in the shPOLE2 group after 26 days of observation (Fig. 4B). The comparison of tumor weight between shPOLE2 group and shCtrl group showed that the reduction of POLE2 weakened tumor growth more intuitively (Fig. 4C), which can be observed from Fig. 4D. Besides, Ki67 is a well-known proliferation marker for the evaluation of cell proliferation [30, 31]. IHC staining analysis of mice tumor tissues showed that the signal intensity of Ki67 in shPOLE2 group was significantly weaker than that in control group (Fig. 4E), which further confirmed that POLE2 knockdown could inhibit the tumor formation. In view of the above results, downregulation of POLE2 might suppress tumor growth in vivo.

### POLE2 promotes AURKA-mediated FOXM1 de-ubiquitination

The potential molecular mechanism of POLE2 involved in GBM cells was preliminarily explored in this study. The downstream molecular mechanism of POLE2 on the regulation of GBM cells was analyzed through human Gene Chip. The results showed that knockdown of POLE2 resulted in upregulation of 1983 DEGs and downregulation of 1383 DEGs (Fig. S2A). IPA-based analysis of the disease and function (Fig. S2B) as well as canonical pathway (Fig. S2C) showed that these DEGs were enriched in signaling pathways associated with cell proliferation and death. In addition, the most significant DEGs were selected by PCR (Fig. S2D) and verified by WB (Fig. 2E). Aurora kinase A (AURKA) was preliminarily considered as a downstream target of POLE2 in GBM cells.

**Fig. 5:**
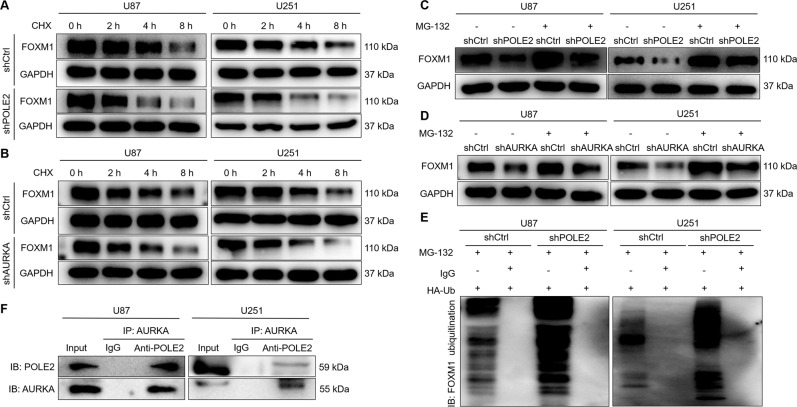
POLE2 knockdown regulates AURKA-mediated FOXM1 ubiquitination. **A**–**B** The protein stability of FOXM1 in U87 and U251 cells after POLE2 knockdown (**A**) or AURKA knockdown (**B**) was examined. **C**–**D** After treatment with MG-132, levels of FOXM1 proteins in U87 and U251 cells with POLE2 (**C**) or AURKA (**D**) knockdown was examined. **E** The lysates of U87 and U251 cells were immunoprecipitated and WB was performed to examine the ubiquitination of FOXM1. **F** Co-IP analysis of interaction of POLE2 and FOXM1 in U87 and U251 cells. The representative images were selected from at least three independent experiments.

In view the fact that AURKA could directly bind and attenuate ubiquitination of Forkhead Box M1 (FOXM1) [32], which has been shown to promote the progression of GBM [33]. In addition, analysis based on the TCGA database shows that the mRNA expression level of AURKA and FOXM1 is abundantly expressed in GBM (Fig. S3A–B). Although there is no significant correlation between the expression level of AURKA and the survival of GBM patients, GBM patients with high AURKA expression have a high probability of short survival (Fig. S3C). Moreover, the relationship between the expression level of FOXM1 and the survival time of GBM patients shows the same situation (Fig. S3D). Accordingly, we hypothesized that POLE2 regulated GBM through AURKA-mediated FOXM1 ubiquitination. In order to verify our hypothesis, we carried out the following experiment. After treatment of protein synthesis inhibitor CHX (0.2 mg/mL), we examined the protein stability of FOXM1 in U87 and U251 cells after POLE2 knockdown or AURKA knockdown, respectively (Fig. 5A, B). The results showed that decreased expression of POLE2 led to the weakening of FOXM1 protein stability in GBM cells (Fig. 5A). Similarly, AURKA could affect the protein stability of FOXM1 (Fig. 5B). Interestingly, the addition of proteasome inhibitor MG-132 (20 μM) partially eliminated the effect of POLE2 or AURKA knockdown on FOXM1 protein stability in GBM cells (Fig. 5C, D), indicating the involvement of proteasome in POLE2-induced regulation of FOXM1. Subsequently, we evaluated the regulation of POLE2 on FOXM1 ubiquitination, and the results showed that knockdown of POLE2 significantly promoted FOXM1 ubiquitination (Fig. 5E), thus decreasing FOXM1 stability. Considering that previous study demonstrated that AURKA attenuated ubiquitination to stabilize FOXM1 [32], we further explored the interaction between POLE2 and AURKA (Fig. 5F). As expected, there was an interaction between AURKA and POLE2. Taken together, POLE2 may promote GBM through AURKA-mediated de-ubiquitination of FOXM1.

### Downregulation of FOXM1 reverses the promotion of POLE2 on the malignant phenotype of GBM cells

**Fig. 6:**
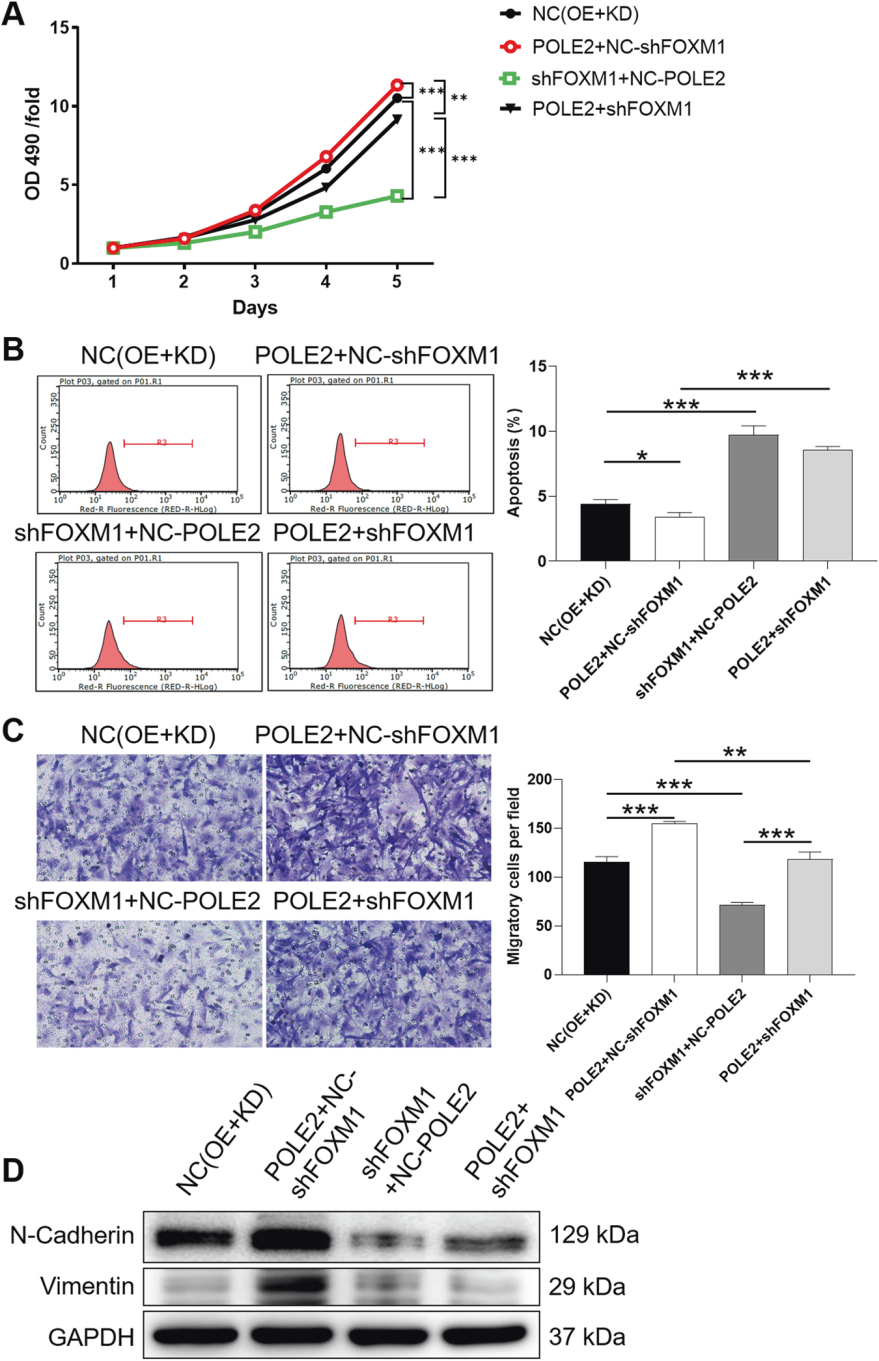
Downregulation of FOXM1 weakens the promoting effects of POLE2 on GBM. U251 cells were subjected to the detection of viability (**A**), apoptosis (**B**), migration (**C**), EMT marker expression (**D**). Notably, NC(OE + KD) was the cells transfected with the empty plasmid, as negative control; POLE2 + NC-shFOXM1 was the cells transfected with lentivirus POLE2 and NC-shFOXM1 for upregulating POLE2; shFOXM1+NC-POLE2 was the cells transfected with lentivirus shFOXM1 and NC-POLE2 for downregulating FOXM1; POLE2 + shFOXM1 was the cells transfected with lentivirus POLE2 and shFOXM1 for upregulating POLE2 and downregulating FOXM1. The representative images were selected from at least three independent experiments. Data were shown as mean ± SD. *P < 0.05, **P < 0.01, ***P < 0.001.

To fully verify the effects of POLE2, AURKA, and FOXM1 in GBM, the functional recovery assays was conducted. We used lentivirus shFOXM1 or shAURKA to interfere with GBM cells, respectively, and constructed U251 cells with FOXM1 (Fig. S4A–D) and AURKA low expression (Fig. S4E–H). The loss-of-function assays demonstrated that knockdown of AURKA in U251 cells showed a significant inhibitory effect on the biological malignancies, which was exacerbated by the simultaneous downregulation of AURKA and POLE2 (Fig. S5A–E). Analogously, we established U251 cells overexpressing POLE2 (POLE2 + NC-shFOXM1), simultaneously upregulating POLE2 and downregulating FOXM1 (POLE2 + shFOXM1), respectively. Notably, NC(OE + KD) was the cells transfected with empty plasmid, as negative control; shFOXM1+NC-POLE2 was the cells transfected with lentivirus shFOXM1 and NC-POLE2 for downregulating FOXM1. Knockdown of FOXM1 could inhibit biological behaviors of U251 cells on slowing down of proliferation (*P* < 0.001) (Fig. 6A), enhancement of apoptosis (*P* < 0.001) (Fig. 6B), and weakening of migration (*P* < 0.001) (Fig. 6C) and EMT (Fig. 6D). As expected, overexpression of POLE2 significantly promoted the malignant behavior of U251 cells, including increased proliferation, decreased apoptosis rate, enhanced migration, and EMT (Fig. 6A–D). Furthermore, we found that downregulation of FOXM1 could partially recover the promoting effects of POLE2 on GBM cells (Fig. 6A–D).

## Discussion

In recent years, various small molecules and signaling pathways involved in the regulation of GBM biological behaviors have been extensively investigated. Therefore, it is necessary to thoroughly explore the molecular mechanism of GBM to identify more effective molecular targets for GBM. In this study, the biological function of POLE2 in GBM was explored. A significant finding of this study was the discovery of a promoting role of POLE2 in GBM. We found that POLE2 was highly expressed in GBM. Furthermore, knockdown of POLE2 could inhibit the biological behaviors of GBM in vitro and in vivo. Specifically, CENPO knockdown inhibited cell proliferation, enhanced cell sensitivity, weakened migration, and EMT of U251 cells.

On the other hand, FOXM1 was regarded as a downstream target of POLE2 in the regulation of GBM. FOXM1 is a transcription factor of the Forkhead box (Fox) protein superfamily [34]. FOXM1 is an important component of a wide range of biological activities, including maintenance of mitotic spindle integrity, regulation of cell cycle, angiogenesis, metastasis, apoptosis, DNA damage repair, and tissue regeneration [35–37]. In addition, FOXM1 has been identified as one of the most DEGs in most solid tumors [38]. Numerous evidences indicate that FOXM1 expression is increased in a variety of human cancers. Lee et al. clarified that dual inhibition of FOXM1 and its compensatory signaling pathway decreased the survival of ovarian cancer cells [39]. In addition, FOXM1 facilitates breast cancer cell stemness and migration [40]. Moreover, FOXM1 has been shown to promote the progression of GBM [33]. Interestingly, FOXM1 can recruit AURKA as a cofactor to activate FOXM1 target genes in a kinase-independent manner. Besides, AURKA and FOXM1 inhibition by either genetic knockdown or pharmacologic inhibitors impair melanoma growth and survival [41]. AURKA and FOXM1 participate in a tightly coupled positive feedback loop to enhance the BCSC phenotype [42]. However, the underlying mechanism of AURKA and FOXM1 in GBM remains inclusive.

Previous study demonstrated that AURKA could directly bind and attenuate the ubiquitin of FOXM1 [32]. Ubiquitination is a widespread post-translational modification that mediates the localization, metabolism, function, regulation, and degradation of proteins in cells [43]. Moreover, ubiquitination plays a central role in the onset of cancers and cardiovascular diseases [44]. AURKA directly binds and attenuates the ubiquitin of FOXM1, which enhances paclitaxel resistance in triple‐negative breast cancer [32]. Here, we demonstrated that POLE2 regulated AURKA-mediated FOXM1 ubiquitination in GBM. Furthermore, downregulation of FOXM1 could partially reverse the promoting effect of POLE2 overexpression on GBM. In conclusion, POLE2 promoted the biological behaviors of GBM through promoting AURKA-mediated stabilization of FOXM1, which may provide the theoretical basis of molecular therapy for GBM.

## Conclusion

A significant finding of this study was the discovery of a promoting role of POLE2 in human GBM. We identified that POLE2 was highly expressed in GBM. Knockdown of POLE2 knockdown could inhibit the malignant behaviors of GBM in vitro and in vivo. POLE2 facilitated the biological behaviors of GBM through promoting AURKA-mediated stabilization of FOXM1. However, there were still some shortcomings in this study. It was worth mentioning that the clinical sample size was small and only had reference value. Secondly, the regulatory role of POLE2 and AURKA had not been clearly elucidated, which required further exploration.

## Supplementary information

Supplementary figure legends

Table S1

Table S2

Fig S1

Fig S2

Fig S3

Fig S4

Fig S5

aj-checklist

## Data Availability

The data used and analyzed during the current study are available from the corresponding author on reasonable request.

## References

[1] McFaline-Figueroa JR, Lee EQ (2018). Brain tumors. Am J Med.

[2] Chen R, Smith-Cohn M, Cohen AL, Colman H (2017). Glioma subclassifications and their clinical significance. Neurotherapeutics..

[3] Gusyatiner O, Hegi ME (2018). Glioma epigenetics: from subclassification to novel treatment options. Semin Cancer Biol.

[4] Bush NA, Chang SM, Berger MS (2017). Current and future strategies for treatment of glioma. Neurosurg Rev.

[5] McKhann GM, Duffau H (2019). Low-grade glioma: epidemiology, pathophysiology, clinical features, and treatment. Neurosurg Clin N. Am.

[6] Xiong L, Wang F, Qi Xie X (2019). Advanced treatment in high-grade gliomas. J BUON.

[7] Siegel RL, Miller KD, Jemal A (2020). Cancer statistics, 2020. CA Cancer J Clin.

[8] Rajaratnam V, Islam MM, Yang M, Slaby R, Ramirez HM, Mirza SP. Glioblastoma: pathogenesis and current status of chemotherapy and other novel treatments. Cancers. 2020;12:937. 10.3390/cancers12040937 PMC7226351 32290213

[9] Zhou Q, Effati R, Talvinen K, Pospiech H, Syvaoja JE, Collan Y (2008). Genomic changes of the 55 kDa subunit of DNA polymerase epsilon in human breast cancer. Cancer Genomics Proteom.

[10] Foiani M, Marini F, Gamba D, Lucchini G, Plevani P (1994). The B subunit of the DNA polymerase alpha-primase complex in Saccharomyces cerevisiae executes an essential function at the initial stage of DNA replication. Mol Cell Biol.

[11] Loeb LA, Monnat RJ (2008). DNA polymerases and human disease. Nat Rev Genet.

[12] Briggs S, Tomlinson I (2013). Germline and somatic polymerase epsilon and delta mutations define a new class of hypermutated colorectal and endometrial cancers. J Pathol.

[13] Yoshida R, Miyashita K, Inoue M, Shimamoto A, Yan Z, Egashira A (2011). Concurrent genetic alterations in DNA polymerase proofreading and mismatch repair in human colorectal cancer. Eur J Hum Genet.

[14] Church DN, Briggs SE, Palles C, Domingo E, Kearsey SJ, Grimes JM (2013). DNA polymerase epsilon and delta exonuclease domain mutations in endometrial cancer. Hum Mol Genet.

[15] Hartmann E, Fernandez V, Moreno V, Valls J, Hernandez L, Bosch F (2008). Five-gene model to predict survival in mantle-cell lymphoma using frozen or formalin-fixed, paraffin-embedded tissue. J Clin Oncol.

[16] Liu D, Zhang XX, Xi BX, Wan DY, Li L, Zhou J (2014). Sine oculis homeobox homolog 1 promotes DNA replication and cell proliferation in cervical cancer. Int J Oncol.

[17] Zekri AR, Hassan ZK, Bahnassy AA, Khaled HM, El-Rouby MN, Haggag RM (2015). Differentially expressed genes in metastatic advanced Egyptian bladder cancer. Asian Pac J Cancer Prev.

[18] Li J, Wang J, Yu J, Zhao Y, Dong Y, Fan Y (2018). Knockdown of POLE2 expression suppresses lung adenocarcinoma cell malignant phenotypes in vitro. Oncol Rep..

[19] Pearlman A, Rahman MT, Upadhyay K, Loke J, Ostrer H (2019). Ectopic Otoconin 90 expression in triple negative breast cancer cell lines is associated with metastasis functions. PLoS ONE.

[20] Rogers RF, Walton MI, Cherry DL, Collins I, Clarke PA, Garrett MD (2020). CHK1 inhibition is synthetically lethal with loss of B-family DNA polymerase function in human lung and colorectal cancer cells. Cancer Res.

[21] Wu Z, Wang YM, Dai Y, Chen LA (2020). POLE2 serves as a prognostic biomarker and is associated with immune infiltration in squamous cell lung cancer. Med Sci Monit.

[22] Zhu Y, Chen G, Song Y, Chen Z, Chen X (2020). POLE2 knockdown reduce tumorigenesis in esophageal squamous cells. Cancer Cell Int.

[23] Beckman G, Beckman L, Ponten J, Westermark B (1971). G-6-PD and PGM phenotypes of 16 continuous human tumor cell lines. Evidence against cross-contamination and contamination by HeLa cells. Hum Hered.

[24] Ponten J, Macintyre EH (1968). Long term culture of normal and neoplastic human glia. Acta Pathol Microbiol Scand.

[25] Lin JS, Lai EM (2017). Protein-protein interactions: co-immunoprecipitation. Methods Mol Biol.

[26] Li L, Li W (2015). Epithelial-mesenchymal transition in human cancer: comprehensive reprogramming of metabolism, epigenetics, and differentiation. Pharm Ther.

[27] Yeung KT, Yang J (2017). Epithelial-mesenchymal transition in tumor metastasis. Mol Oncol.

[28] Kishore C, Sundaram S, Karunagaran D (2019). Vitamin K3 (menadione) suppresses epithelial-mesenchymal-transition and Wnt signaling pathway in human colorectal cancer cells. Chem Biol Interact.

[29] Li B, Cheng J, Wang H, Zhao S, Zhu H, Li C (2019). CCNB1 affects cavernous sinus invasion in pituitary adenomas through the epithelial-mesenchymal transition. J Transl Med.

[30] Yang C, Zhang J, Ding M, Xu K, Li L, Mao L (2018). Ki67 targeted strategies for cancer therapy. Clin Transl Oncol.

[31] Menon SS, Guruvayoorappan C, Sakthivel KM, Rasmi RR (2019). Ki-67 protein as a tumour proliferation marker. Clin Chim Acta.

[32] Yang N, Wang C, Wang J, Wang Z, Huang D, Yan M (2019). Aurora kinase A stabilizes FOXM1 to enhance paclitaxel resistance in triple-negative breast cancer. J Cell Mol Med.

[33] Zhang C, Han X, Xu X, Zhou Z, Chen X, Tang Y (2018). FoxM1 drives ADAM17/EGFR activation loop to promote mesenchymal transition in glioblastoma. Cell Death Dis.

[34] Clark KL, Halay ED, Lai E, Burley SK (1993). Co-crystal structure of the HNF-3/fork head DNA-recognition motif resembles histone H5. Nature..

[35] Costa RH (2005). FoxM1 dances with mitosis. Nat Cell Biol.

[36] Laoukili J, Kooistra MR, Bras A, Kauw J, Kerkhoven RM, Morrison A (2005). FoxM1 is required for execution of the mitotic programme and chromosome stability. Nat Cell Biol.

[37] Wonsey DR, Follettie MT (2005). Loss of the forkhead transcription factor FoxM1 causes centrosome amplification and mitotic catastrophe. Cancer Res.

[38] Okabe H, Satoh S, Kato T, Kitahara O, Yanagawa R, Yamaoka Y (2001). Genome-wide analysis of gene expression in human hepatocellular carcinomas using cDNA microarray: identification of genes involved in viral carcinogenesis and tumor progression. Cancer Res.

[39] Lee DW, Lee W, Kwon M, Lee HN (2021). Dual inhibition of FOXM1 and its compensatory signaling pathway decreased the survival of ovarian cancer cells. Oncol Rep..

[40] Sun HL, Men JR, Liu HY, Liu MY, Zhang HS (2020). FOXM1 facilitates breast cancer cell stemness and migration in YAP1-dependent manner. Arch Biochem Biophys.

[41] Puig-Butille JA, Vinyals A, Ferreres JR, Aguilera P, Cabre E, Tell-Marti G (2017). AURKA overexpression is driven by FOXM1 and MAPK/ERK activation in melanoma cells harboring BRAF or NRAS mutations: impact on melanoma prognosis and therapy. J Invest Dermatol.

[42] Yang N, Wang C, Wang Z, Zona S, Lin SX, Wang X (2017). FOXM1 recruits nuclear Aurora kinase A to participate in a positive feedback loop essential for the self-renewal of breast cancer stem cells. Oncogene..

[43] Nakamura N. Ubiquitin System. Int J Mol Sci. 2018;19:1080. 10.3390/ijms19041080 PMC5979459 29617326

[44] Popovic D, Vucic D, Dikic I (2014). Ubiquitination in disease pathogenesis and treatment. Nat Med.

