# Supplementary material for: POLE2 facilitates the malignant phenotypes of glioblastoma through promoting AURKA-mediated stabilization of FOXM1
Source: Cell Death Dis. 2022 Jan 17;13(1):61. doi: 10.1038/s41419-021-04498-7 (PMC8763902; doi:10.1038/s41419-021-04498-7)
Supplement: Supplementary file 1 — Supplementary figure legends [file 41419_2021_4498_MOESM1_ESM.docx]

**Supplementary legend**

**Fig S1.** (A) The correlation analysis between POLE2 expression and survival probability of patients with GBM was analyzed Based on the TCGA-GBM database. (B) The infection efficiency of shCtrl, shPOLE2 in U87 and U251 cells was evaluated through observing the fluorescence of GFP on lentivirus vector. (C-D) The knockdown efficiencies of shCtrl, shPOLE2 in U87 and U251 cells were accessed by qPCR (C) and WB (D). The representative images were selected from at least 3 independent experiments. Data was shown as mean ± SD. **P < 0.01, ***P < 0.001.

**Fig S2.** (A) A Prime View Human Gene Expression Array was performed to identify the differentially expressed genes (DEGs) between shPOLE2 and shCtrl groups of U251 cells. (B) The enrichment of the DEGs in IPA disease and function was analyzed by IPA. (C) The enrichment of the DEGs in canonical signaling pathways was analyzed by IPA. (D) qPCR was used to detect the expression of several selected DEGs in U251 cells with or without POLE2 knockdown. (E) WB was used to detect the most significant DEGs in U251 cells with or without POLE2 knockdown.

**Fig S3.** (A-B) The mRNA expression of AURKA (A) and FOXM1 (B) in tumor samples (169 cases) and normal samples (5 cases) of GBM patients was analyzed based on the Cancer Genome Atlas (TCGA) database. (C-D) The correlation analysis between AURKA (C) and FOXM1 (D) expression and survival probability of patients with GBM was analyzed Based on the TCGA-GBM database.

**Fig S4.** (A) The knockdown efficiency of 3 shRNAs designed for FOXM1 knockdown were evaluated by qPCR. (B-D) The expression of FOXM1 or POLE2 in U251 cells were accessed by qPCR (B, C) and WB (D). (E) The knockdown efficiency of 3 shRNAs designed for AURKA knockdown were evaluated by qPCR. (F-H) The expression of AURKA or POLE2 in U251 cells were accessed by qPCR (F, G) and WB (H).

**Fig S5.** U251 cells were subjected to the detection of proliferation (A), apoptosis (B), cell cycle (C) and migration (D-E). Notably, shCtrl was the cells transfected with empty plasmid, as negative control; shAURKA was the cells that downregulating AURKA. shAURKA+shPOLE2 was the cells that simultaneously downregulating AURKA and POLE2.
