# Supplementary material for: POLE2 facilitates the malignant phenotypes of glioblastoma through promoting AURKA-mediated stabilization of FOXM1
Source: Cell Death Dis. 2022 Jan 17;13(1):61. doi: 10.1038/s41419-021-04498-7 (PMC8763902; doi:10.1038/s41419-021-04498-7)
Supplement: Supplementary file 2 — Table S1 [file 41419_2021_4498_MOESM2_ESM.docx]

Table S1. Antibodies used in IHC and WB.

| Primary antibody | Dilution in IHC | Source species | Company | Catalog No. |
| --- | --- | --- | --- | --- |
| POLE2 | 1:100 | Rabbit | BIOSS | bs-14356R |
| AURKA | 1:100 | Rabbit | Abcam | ab52973 |
| Ki67 | 1:200 | Rabbit | Abcam | ab16667 |
| Primary antibody | Dilution in WB | Source species | Company | Catalog No. |
| POLE2 | 1:1000 | Rabbit | Abcam | ab180214 |
| N-cadherin | 1:1000 | Rabbit | Abcam | ab18203 |
| Vimentin | 1:1000 | Rabbit | Abcam | ab92547 |
| Snail | 1:1000 | Rabbit | CST | 3879S |
| Akt | 1:1000 | Rabbit | CST | 4685 |
| p-Akt | 1:500 | Rabbit | CST | 4685 |
| CCND1 | 1:2000 | Rabbit | CST | 2978 |
| CCNB1 | 1:3000 | Mouse | Abcam | ab72 |
| PIK3CA | 1:1000 | Rabbit | Abcam | ab40776 |
| Bcl-2 | 1:2000 | Rabbit | CST | ab182858 |
| HSP27 | 1:1000 | Rabbit | CST | ab109376 |
| HSP70 | 1:1000 | Mouse | CST | 46477S |
| Survivin | 1:2000 | Rabbit | CST | 10508-1-AP |
| AR | 1:1000 | Rabbit | Abcam | ab74272 |
| AURKA | 1:1000 | Rabbit | Abcam | ab52973 |
| CD44 | 1:2000 | Rabbit | Abcam | ab157107 |
| CEBPA | 1:1000 | Rabbit | Abcam | ab40764 |
| GAPDH | 1:3000 | Rabbit | Bioworld | AP0063 |
| Secondary antibody | Dilution |  | Company | Catalog No. |
| HRP Goat Anti-Rabbit IgG (IHC) | 1:400 |  | Abcam | ab6721 |
| HRP Goat Anti-Mouse IgG (WB) | 1:3000 |  | Beyotime | A0216 |
| HRP Goat Anti- Rabbit IgG (WB) | 1:3000 |  | Beyotime | A0208 |
|  |  |  |  |  |
