# Supplementary material for: POLE2 facilitates the malignant phenotypes of glioblastoma through promoting AURKA-mediated stabilization of FOXM1
Source: Cell Death Dis. 2022 Jan 17;13(1):61. doi: 10.1038/s41419-021-04498-7 (PMC8763902; doi:10.1038/s41419-021-04498-7)
Supplement: Supplementary file 3 — Table S2 [file 41419_2021_4498_MOESM3_ESM.docx]

Table S2. Primers used in qPCR

| Primer Name | Upstream Primer  Sequence (5’-3’) | Downstream Primer  Sequence (5’-3’) | Fragment Size (bp) |
| --- | --- | --- | --- |
| POLE2 | TGAGAAGCAACCCTTGTCATC | TCATCAACAGACTGACTGCATTC | 84 |
| CEBPA | TGCTCCCACAAATGAAGCCT | GCCTCCGCTAACCAGGATTT | 127 |
| CDC27 | TTCACCTGATACTGTCCCACTG | GCTGCTGGTCCTCCTAATAAACT | 102 |
| G3BP1 | CTGAAATCCAAGAGGAAAAGCC | GTCACAGATGCCCAAGAAAATG | 142 |
| RAD17 | AGTATGTGAGGATTGGTCGATGTC | TTGTTGGTGCCACAGGGTT | 147 |
| CD44 | TGGGTTCATAGAAGGGCACG | ATACTGGGAGGTGTTGGATGTG | 106 |
| IL6 | GGTACATCCTCGACGGCATCT | GTGCCTCTTTGCTGCTTTCAC | 81 |
| CDC20 | AATGGAGCAGCCTGGGGAATA | CGGGCAGAGTGACTGGTCATAT | 147 |
| KCTD3 | CAGCCCTATGCTTTTCAGATTC | CAGGCTGTCGTTCATTCCAT | 107 |
| PIK3R1 | CAGCCCTATGCTTTTCAGATTC | TCTTTCTTCCAACTGGGCATC | 92 |
| EGLN3 | TTCCTCCTGTCCCTCATCGA | GCAAGCCACCATTGCCTTAG | 96 |
| PDGFA | AGACCAGGACGGTCATTTACG | ACTTGACACTGCTCGTGTTGC | 129 |
| TWIST1 | CCGGAGACCTAGATGTCATTG | CCACGCCCTGTTTCTTTGA | 149 |
| AURKA | GGCACCTGAAAATAATCCTGAG | CAAAGTCTTCCAAAGCCCACT | 89 |
| AGFG1 | GTCTGTAAACCAGCTTTGTTCG | CCAGTGCTGATTTTGGAACC | 94 |
| FBN1 | ACACTCCTCGCATTCCTCAG | ATATCCGCCTGGAAACCTG | 134 |
| AR | TGAGGAGACAACCCAGAAGC | ACTACACCTGGCTCAATGGC | 99 |
| BCOR | CAACTGGAATCAAGGGAAGACT | AAAGCTGGGTTTGGACACG | 112 |
| FAM189B | CGTCTGTGGGCTCACCATTT | GGTCCAGGGAGAAGATTTGG | 149 |
| FOXO3 | AGTCGGACCCCTTGATGTCT | GGTGGAGCAAGTTCTGATTGA | 144 |
| GAPDH | TGACTTCAACAGCGACACCCA | CACCCTGTTGCTGTAGCCAAA | 121 |
