# Supplementary figures and images for: POLE2 facilitates the malignant phenotypes of glioblastoma through promoting AURKA-mediated stabilization of FOXM1
Source: Cell Death Dis. 2022 Jan 17;13(1):61. doi: 10.1038/s41419-021-04498-7 (PMC8763902; doi:10.1038/s41419-021-04498-7)

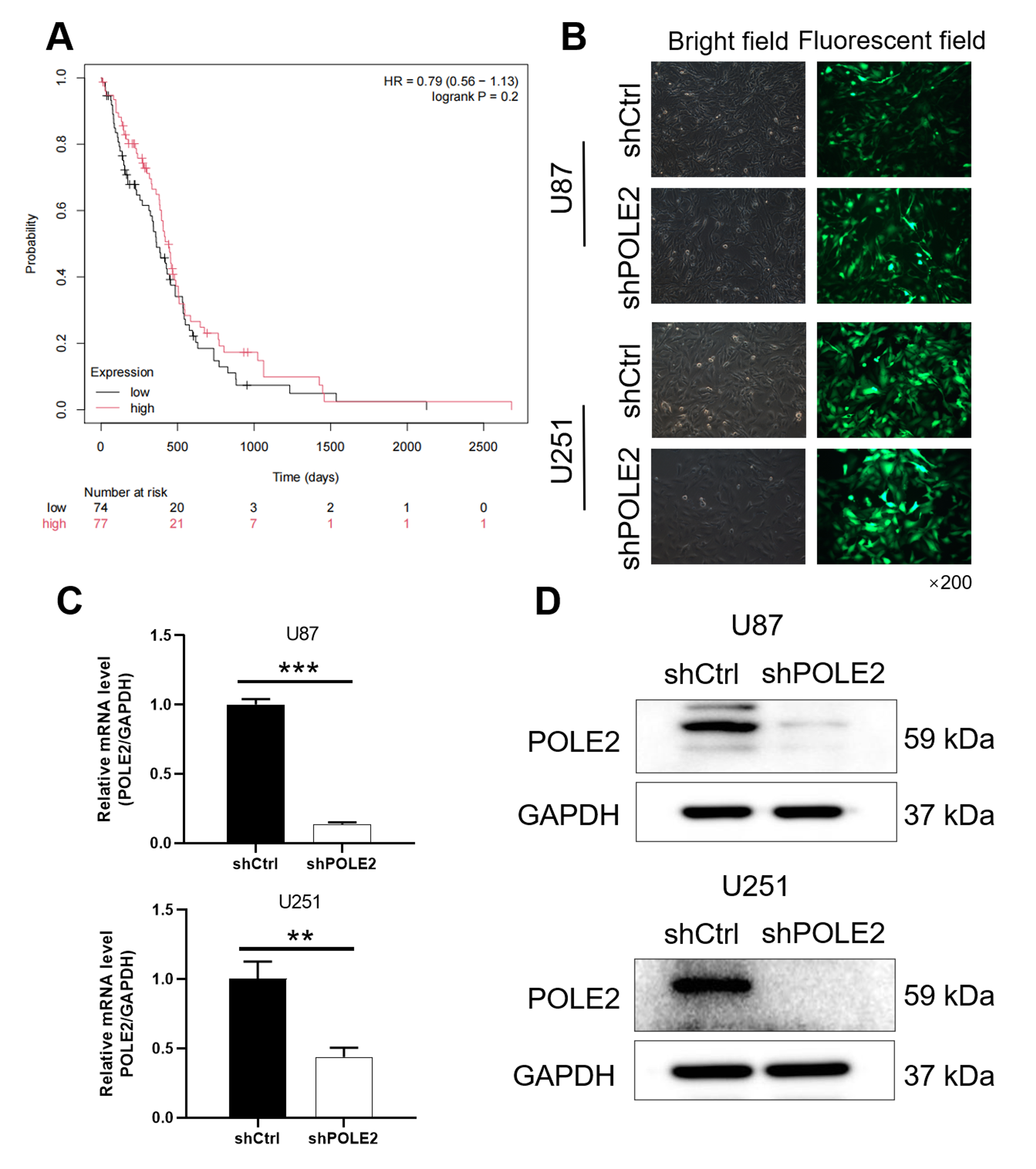

Supplement: Supplementary file 4 — Fig S1 [file 41419_2021_4498_MOESM4_ESM.tif]

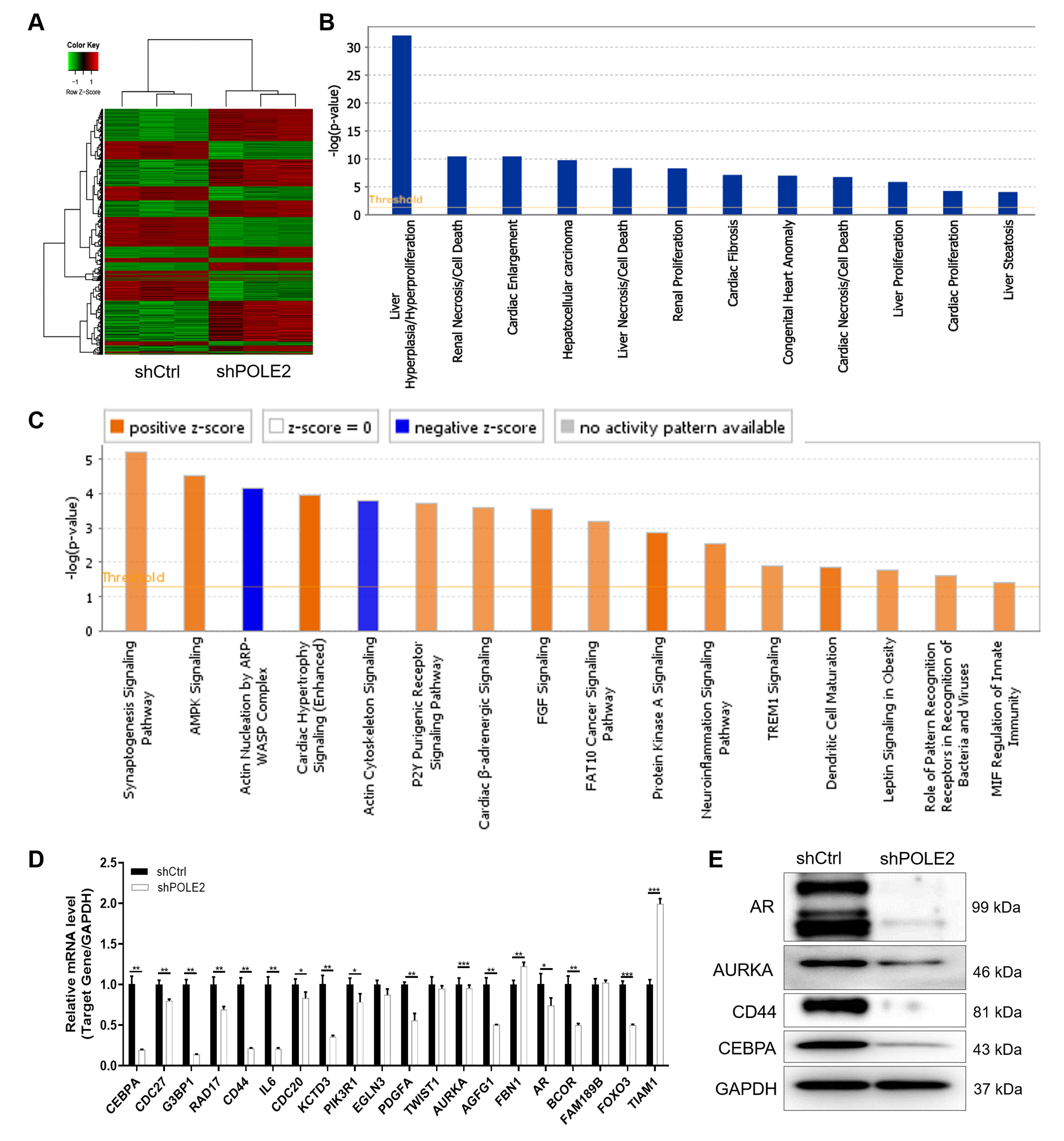

Supplement: Supplementary file 5 — Fig S2 [file 41419_2021_4498_MOESM5_ESM.tif]

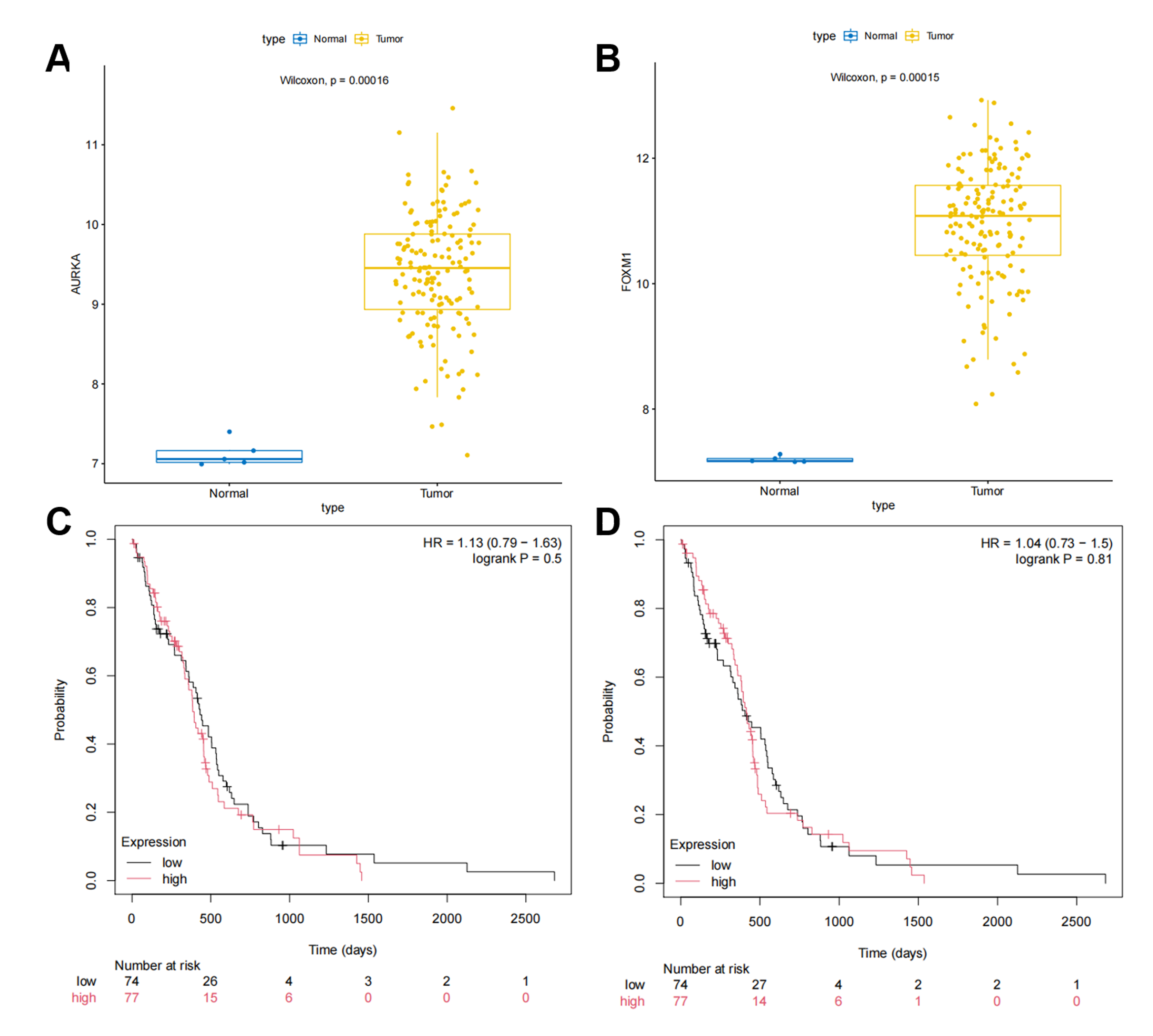

Supplement: Supplementary file 6 — Fig S3 [file 41419_2021_4498_MOESM6_ESM.tif]

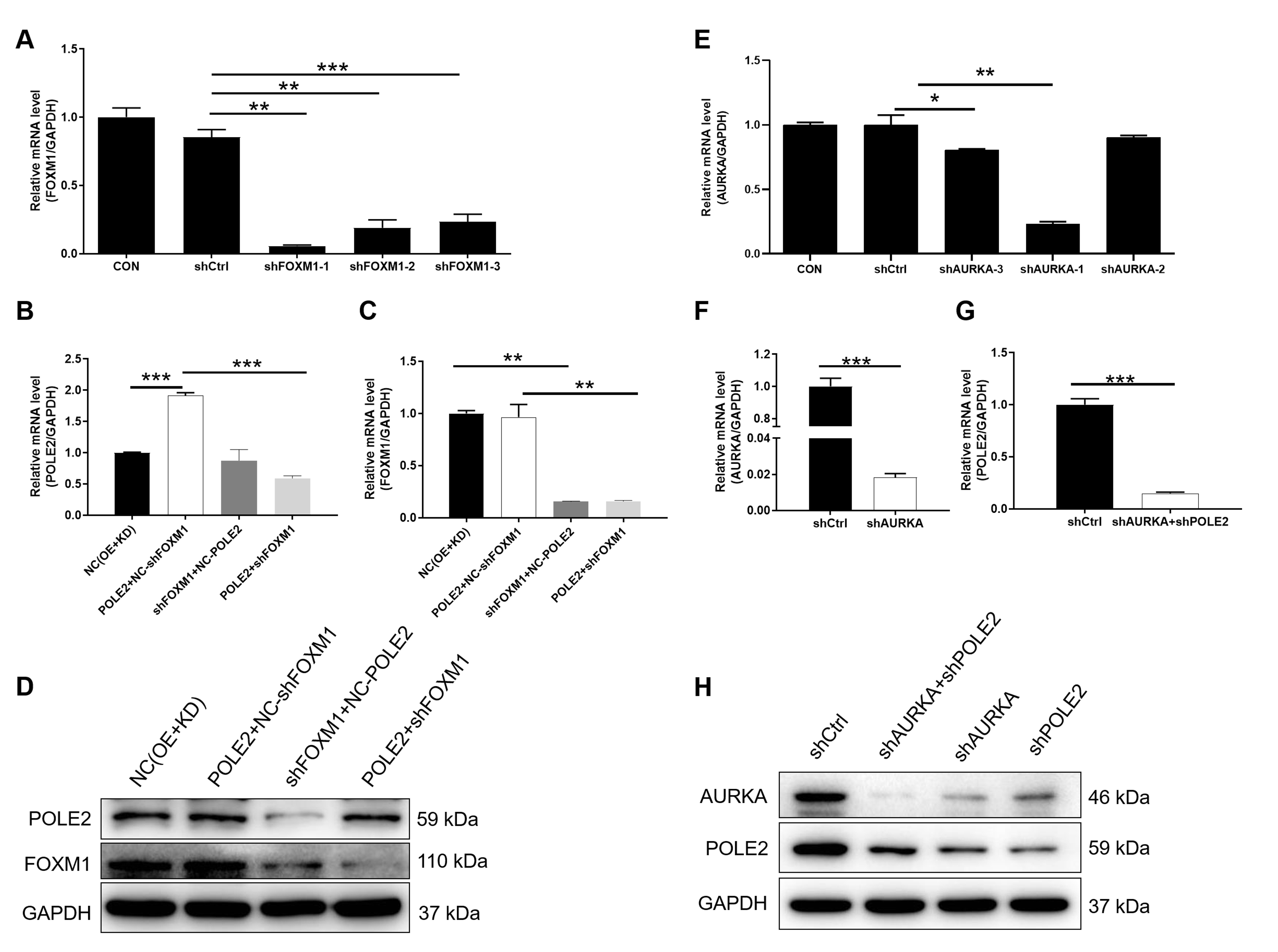

Supplement: Supplementary file 7 — Fig S4 [file 41419_2021_4498_MOESM7_ESM.tif]

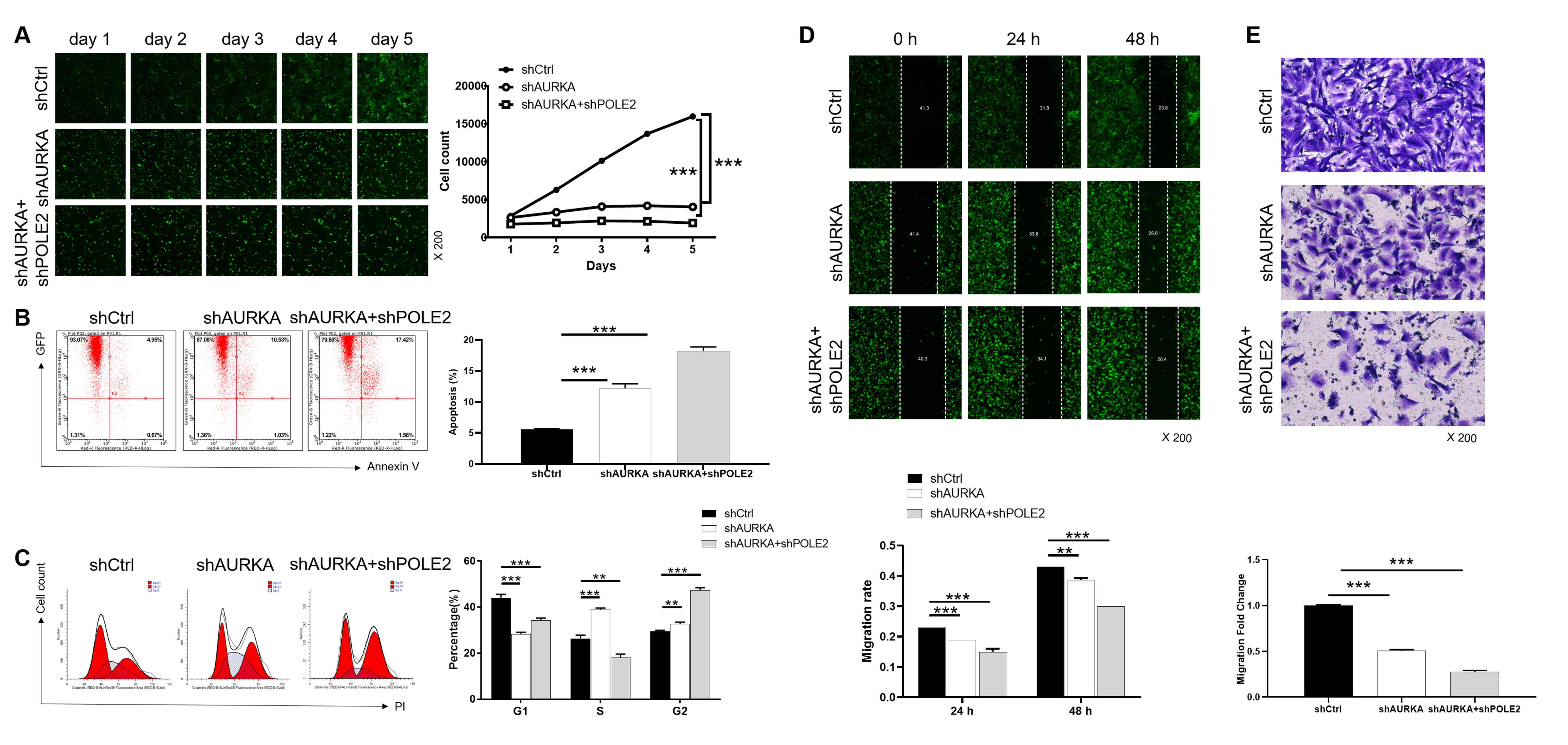

Supplement: Supplementary file 8 — Fig S5 [file 41419_2021_4498_MOESM8_ESM.tif]
